# The More (Social Group Memberships), the Merrier: Is This the Case for Asians?

**DOI:** 10.3389/fpsyg.2016.01001

**Published:** 2016-07-12

**Authors:** Melissa X.-L. Chang, Jolanda Jetten, Tegan Cruwys, Catherine Haslam, Nurul Praharso

**Affiliations:** School of Psychology, The University of QueenslandBrisbane, QLD, Australia

**Keywords:** multiple group membership, social support, well-being, culture, social identity

## Abstract

While previous studies have consistently shown that belonging to multiple groups enhances well-being, the current research proposes that for Asians, multiple group memberships (MGM) may confer fewer well-being benefits. We suggest that this is due, in part, to Asian norms about relationships and support seeking, making Asians more reluctant to enlist social support due to concerns about burdening others. Overall, MGM was associated with enhanced well-being in Westerners (Study 2), but not Asians (Studies 1–3). Study 2 showed that social support mediated the relationship between MGM and well-being for Westerners only. In Study 3, among Asians, MGM benefited the well-being of those who were least reluctant to enlist support. Finally, reviewing the MGM evidence-base to date, relative to Westerners, MGM was less beneficial for the well-being of Asians. The evidence underscores the importance of culture in influencing how likely individuals utilize their group memberships as psychological resources.

## Introduction

We know that social relationships matter for psychological well-being. The research shows that people who belong to more social groups have better psychological well-being than those with fewer social group memberships (Thoits, 1983; Helliwell, 2003; Haslam et al., 2008; Brook et al., 2008; Cruwys et al., 2013; Jetten et al., 2015; Sani et al., 2015). This is due, in part, to social groups providing an important means to access psychological resources such as social support (see Jetten et al., 2014). Social support, in turn, has been found to be associated with mental health benefits that include better adjustment to stressful events (House et al., 1988; Thoits, 1995). It follows logically then, that the more groups an individual belongs to, the more potential social support resources they have at their disposal when encountering challenges or stressors in their life.

Although much of the empirical work on multiple group membership and well-being has generated important insights, these studies have been conducted primarily in Western societies and it remains to be examined whether the findings generalize to non-Western cultures (Henrich et al., 2010). Put simply, will “the more the merrier” effect, in the case of multiple groups, hold for Asians? There are good reasons to believe that such findings may not easily generalize to Asian populations. For example, studies have suggested that cultural norms about relationships in the Asian cultures could make Asians more sensitive to the negative relational consequences of support seeking when compared to European Americans (e.g., burdening others, disrupting group harmony, Taylor et al., 2004; Kim et al., 2006, 2008). To the extent that this applies, multiple group memberships (MGM) may not be associated with greater support seeking in times of difficulty and stress for Asians. These cultural differences in the role that multiple group memberships play raise questions about the nature of its relationship with psychological well-being in Asian cultures. Addressing this issue, the present research examines the role of MGM in Asian and Western contexts.

### Multiple group memberships and well-being—“the more the merrier”

The Social Identity Approach (SIA; Haslam et al., 2009; Jetten et al., 2012, 2014) provides an explanation for the relationship between MGM and well-being. Fundamental to this approach is the idea that social group memberships are critical in initiating a shared sense of identification. When social groups are perceived as meaningful and relevant to characterizing the self, they become psychologically internalized and help to understand the self and one's place in the world (Turner et al., 1987). Hence, social groups have the power to furnish people with a sense of themselves as part of a larger collective (“us”) rather than as merely unique individuals (“I”; Turner, 1982).

Accordingly, when one incorporates groups of others (e.g., one's family, friendship, community, and recreational groups) into one's sense of self, one will feel *psychologically connected* with those others, such that their interests become one's own (Haslam et al., 2012). More importantly still, such internalized group membership provides a meaningful basis to receive and benefit from various forms of social support (Cohen and Wills, 1985; Underwood, 2000; Postmes and Branscombe, 2002; Haslam, 2004; Haslam et al., 2009). This means that it is only when people perceive themselves to share a mutual sense of common group membership with others that they are more likely to give, and be open to receiving, support and other resources from them; facilitating constructive helping between individuals. Applying this logic, social groups should only enhance positive social support and well-being when (and to the extent that) individuals *identify* strongly with them. A large body of empirical work has substantiated this by showing that shared identity is indeed what makes social support possible and effective (Haslam, 2004; Haslam et al., 2005, 2012; Levine et al., 2005). For illustration, in a study by Haslam et al. (2005), it was found that shared group memberships had a positive impact on well-being among hospital patients recovering from heart surgery because such group memberships served as a basis for the receipt of effective support from others.

Following from this, the SIA posits that if group membership serves as a psychological resource (e.g., providing a basis for social support), then it is likely that MGM should enhance this resource (Haslam et al., 2008; Cruwys et al., 2014; Jetten et al., 2015). Individuals who belong to multiple groups are therefore likely to have more potential sources from which to draw social support in times of difficulty and stress, in turn protecting and enhancing their well-being (Iyer et al., 2009; Jetten et al., 2009, 2014). Indeed, several studies have consistently shown that having more group memberships (e.g., belonging to family, friendship, community, recreational groups) is associated with greater psychological well-being.

For example, in one study conducted with individuals who had recently experienced a stroke, Haslam et al. (2008) showed that life satisfaction and well-being were higher for those who belonged to more social groups before their stroke. Furthermore, belonging to multiple groups has also been found to be protective against developing depression (Cruwys et al., 2013), and to be associated with reduced depression and distress among university students and healthy adults during times of important life transitions (Thoits, 1983; Linville, 1985, 1987; Iyer et al., 2009). Along these lines, a longitudinal study with British students entering university showed that having a greater number of group memberships before the move to university predicted better adjustment and well-being once students had been at university for a few months (Iyer et al., 2009). Moreover, there was a clear relationship between the numbers of groups that these students were members of and the amount of social support they reported receiving, such that MGM provided individuals with more sources of support that could be drawn on to provide stability in this period of transition. While the aforementioned studies involved correlational or longitudinal examinations, the beneficial effect of MGM has also been demonstrated experimentally by manipulating the psychological availability of group memberships (i.e., getting participants to think of one, three, or five group memberships; Jones and Jetten, 2011, Study 2).

However, the relationship between MGM and well-being has mostly been demonstrated in Western cultural contexts. This is important because research suggests that individuals in Asian cultural contexts may not utilize their MGM in the same way as individuals in Western cultural contexts. We therefore question the generalizability of the multiple group membership effect and ask in this research whether group memberships play a different role in Asian cultures.

### Multiple group memberships and well-being: a cross-cultural perspective

Research has shown that there is cultural variation in how people construe the self and their relationships with others (e.g., relative emphasis on personal goals or group goals; Markus and Kitayama, 1991), and in turn, their expectations of those relationships. In Western contexts, individualism is emphasized, and individuals are encouraged to promote their uniqueness and act according to their own volition (Heine et al., 1999). By contrast, the emphasis is on collectivism in Asian contexts. Individuals in these contexts are encouraged to maintain closeness and harmony within social groups and view group goals as primary and personal beliefs, needs, and goals as secondary (Markus and Kitayama, 1991; Heine et al., 1999). These differences in cultural norms and expectations about relationships are likely to impact on the extent to which individuals seek social support (Taylor et al., 2004; Kim et al., 2006; Sherman et al., 2009). Specifically, in Asian cultures, individuals are more careful with regards to extracting support from others because they are more concerned about the potential negative relational implications of support seeking (Kim et al., 2008). That is, social support seeking in this cultural context is more likely to be seen as an imposition, burdening the provider of the support because drawing on another person for support can tax that other person's time and attention resources (Seidman et al., 2006). This can also be costly in the sense that it may undermine and disrupt group harmony. In contrast, individuals in Western cultures view social support as personal resources, making them more likely to actively solicit them in times of difficulty (Uchida et al., 2008; Chen et al., 2012).

Evidence supporting this argument comes from Taylor et al. (2004) who showed that Asians were less willing than European Americans to draw on social support from their social networks to cope with stress. Furthermore, they found Asians to be more sensitive to the relational consequences of support seeking than Americans, and that these relational concerns accounted for the cultural differences in the use of social support for coping with stress. This suggests that among Asians, cultural norms (e.g., concerns about burdening others) could discourage the active engagement of one's social support network to cope with stress.

**Figure 1:**
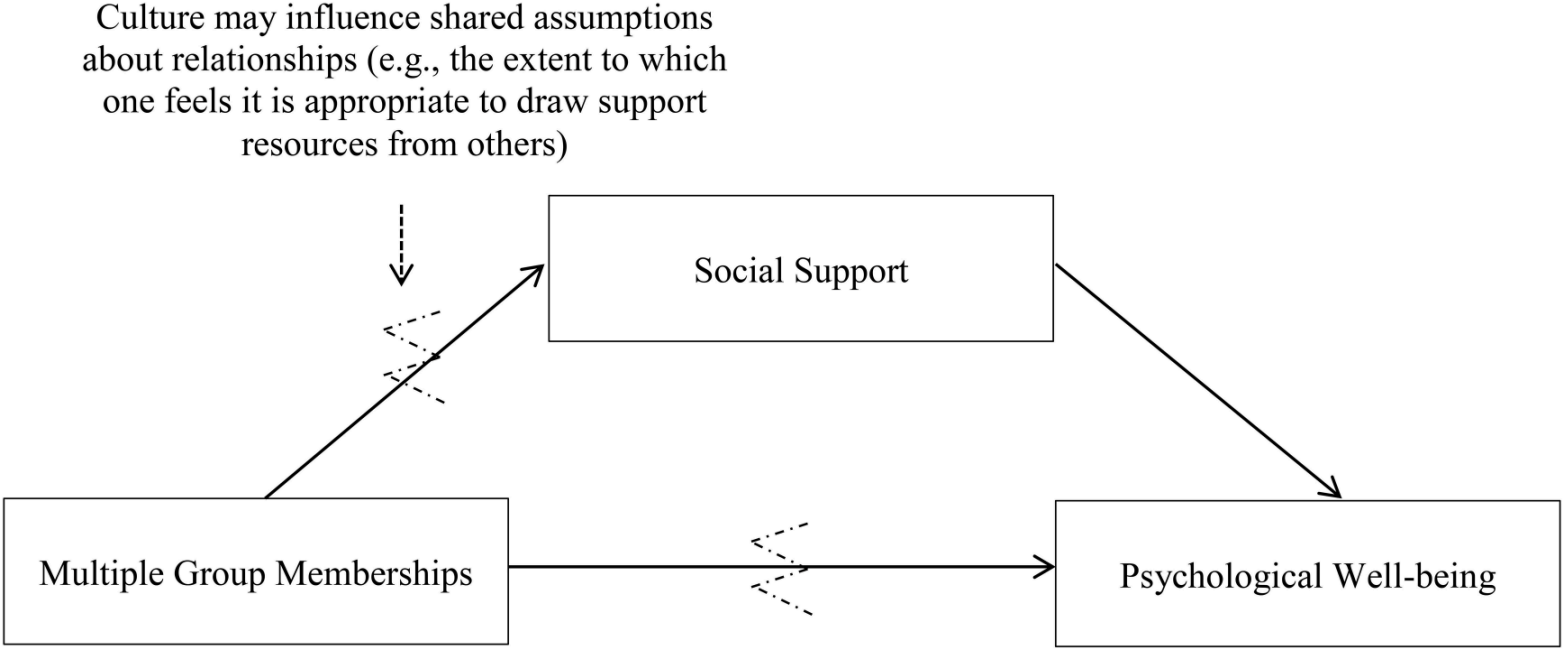
**Proposed relationship between multiple group memberships and well-being, with breaks indicated where we hypothesize the relationship to be weaker among Asians than among Westerners**.

Building on these previous findings, to the extent that MGM are seen as another form of relationship with others (Jetten et al., 2015), it is likely that cultural variations in shared assumptions about relationships and support seeking may influence the way individuals utilize their MGM. Specifically, this may affect the degree to which one feels it is appropriate to draw upon support resources derived from shared group memberships, which would in turn influence one's well-being. Consequently, MGM may confer fewer benefits to the psychological well-being of Asians because cultural norms on relationships and support seeking in Asian cultural contexts might lead to reluctance to tap into social support resources from their group memberships (see Figure 1). Importantly, this line of work extends on previous research examining cultural differences that has focused largely on relationships and support seeking from significant individuals (e.g., a family member, friend; Kim et al., 2008). Here, we consider the particular contribution that relationships and social support from groups of others makes to health and well-being—where group memberships can comprise a range of diverse relationships such as those with family and friendship groups as well as religious, community, and recreational groups. More importantly, we focus on those group memberships that are meaningful and which individuals feel psychologically connected to (i.e., social identification; Jetten et al., 2014).

### The present research

In four studies, we investigated the extent to which cultural normative expectations about support seeking would influence the degree to which individuals derive psychological resources and well-being benefits from belonging to multiple groups. We examined the effect of MGM on a wide range of well-being indicators—notably, life satisfaction, happiness, depression, anxiety, and stress. Study 1 used an exploratory correlational design to examine the role that MGM play in the well-being of Asians. In Studies 2 and 3, we investigated a potential cultural underpinning that might account for why individuals from different cultures (Asian vs. Western) may utilize their group membership resources in different ways. Here, we predicted cross-cultural variation in drawing on support from shared group memberships, such that reluctance to enlist support from relationships may lead to fewer support resources being derived from belonging to multiple groups. Lastly, to examine the robustness of the relationship between MGM and well-being in different cultural contexts, we conducted a review of the empirical literature assessing this cross-sectional relationship (contrasting Asians with Westerners) in Study 4.

## Study 1

In a first exploratory correlational study, we aimed to explore the relationship between MGM and psychological well-being among international Asian students who had just transitioned from their own culture to a Western culture to commence their studies.

Ethical clearance for all studies was provided by the Psychology Ethics committee at the University of Queensland. In all studies, participants ticked a box before starting the survey, indicating their informed consent.

### Method

#### Participants

Participants were 180 international students at a large Australian university who had only been in Australia for 1 month (54 males, 124 females, 2 did not indicate their gender). Participants had a mean age of 22.0 years (*SD* = 4.00) and were born in Asia (i.e., China, Hong Kong, Indonesia, Malaysia, and Singapore).

#### Measures

Participants completed a survey that included demographic questions, and scales measuring multiple group membership and well-being (i.e., life satisfaction, depression, anxiety, stress).

##### Multiple group membership

Multiple group membership was assessed with four items from the Exeter Identity Transition Scale (EXITS; see Haslam et al., 2008). These included “I belong to a lot of groups,” “I join in the activities of lots of different groups,” “I have friends who are members of lots of groups,” and “I have strong ties with lots of different groups.” Each item was rated on a 7-point scale (1 = *Strongly Disagree*, 7 = *Strongly Agree;* α = 0.91).

##### Satisfaction with life scale

Satisfaction with life scale (SWLS; Diener et al., 1985). Five items (e.g., “I am satisfied with my life”) assessed one's judgment of satisfaction with one's life (α = 0.87). Each item was rated on a 7-point scale (1 = *Strongly disagree*, 7 = *Strongly agree*).

##### Depression anxiety stress scales

Depression anxiety stress scales (DASS-21; Lovibond and Lovibond, 1995). Depression, anxiety, and stress were measured using the DASS-21. The DASS-21 consists of three 7-item subscales that measure depression (e.g., I felt down-hearted and blue; α = 0.88), anxiety (e.g., I felt I was close to panic; α = 0.80), and stress (e.g., I found it hard to wind down; α = 0.85). It has excellent reliability and validity in both clinical and non-clinical samples (Henry and Crawford, 2005; Crawford et al., 2009). Participants were asked to indicate how much the items had applied to them during the past week on a scale ranging from 0 = *Did not apply to me at all*, to 3 = *Applied to me very much, or most of the tim*e. For each subscale, responses were summed and multiplied by two in accordance with recommended practice (Lovibond and Lovibond, 1995).

### Results and discussion

Descriptive statistics and bivariate correlations of key variables are presented in Table 1. Multiple group membership was not significantly related to life satisfaction (*r* = 0.121, *p* = 0.105), depression (*r* = −0.104, *p* = 0.167), anxiety (*r* = −0.105, *p* = 0.162), or stress (*r* = −0.070, *p* = 0.350), suggesting that belonging to multiple groups may not be associated with enhanced well-being for Asian participants.

**Table 1:** **Descriptive statistics and correlations**.

**Variable**	***M***	***SD***	**1**	**2**	**3**	**4**	**5**
1. Multiple group membership	3.43	1.29	–	0.12	−0.10	−0.11	−0.07
2. Well-being: Life satisfaction	4.88	1.17		–	−0.36^**^	−0.36^**^	−0.34^**^
3. Well-being: depression	8.94	8.32			–	0.74^**^	0.78^**^
4. Well-being: anxiety	11.68	8.14				–	0.82^**^
5. Well-being: stress	12.43	8.53					–

*Study 1; N = 180*.

^*^p < 0.05,

** *p < 0.01*.

This finding contrasts with mounting evidence of the well-being benefits of MGM (Jetten et al., 2012, 2014), and provides initial support for our argument that Asians may derive fewer well-being benefits from multiple groups relative to Westerners. However, aside from the fact that one should be careful in drawing inferences from an absence of a significant relationship (i.e., confirming the null hypothesis), another limitation is that having only explored this relationship in Asians, questions about (a) the extent to which the multiple group membership effect differs across cultures, and (b) the mechanism underlying this cultural difference (if any), remain. We address these limitations in the following studies.

## Study 2

Study 2 directly compared Western and Asian samples to investigate cultural differences in the relative benefit to psychological well-being of belonging to multiple groups. Our prediction was that culture would moderate the positive effect of MGM on well-being. Specifically, belonging to multiple groups would benefit the well-being of Asians to a lesser extent than Westerners. A second goal of Study 2 was to examine a potential mechanism underlying this cultural difference—the notion that Asians are less likely to derive support resources from their MGM (Taylor et al., 2004; Kim et al., 2006; Sherman et al., 2009). We predicted that only for Western participants, social support would mediate the effect of MGM on psychological well-being. We expected that this mediational path would not be significant for Asian participants. In our examination of these predictions, we adopted happiness and depression as indicators of well-being.

### Method

#### Participants

Participants were 137 undergraduate students at a large Australian university; 60 international students who were born and raised in Asia and described their ethnicity as Chinese (17 males and 43 females), and 77 Australians Caucasians (28 males and 49 females). Participants either received course credit or 10 Australian dollars for their participation. Asian participants (*n* = 60) were on average 23.2 years old (*SD* = 4.01) and the average length of time spent in Australia was 23.73 months. Western participants (*n* = 77) had a mean age of 19.6 years (*SD* = 3.69).

#### Measures

Participants completed a survey that included demographic questions, and scales measuring multiple group membership, social support, and well-being.

##### Multiple group membership

An abbreviated two item scale (i.e., “I am a member of lots of different social groups” and “I have friends who are in lots of different social groups”), assessed the extent to which individuals belong to multiple groups (Jetten et al., 2010; *r* = 0.68). These items have been used in previous research and have been found to demonstrate good internal reliability (see Jetten et al., 2010). Each item was rated on a 7-point scale (1 = *Strongly Disagree*, 7 = *Strongly Agree*).

##### Social support

Participants' level of social support was measured using four items adapted from Van Dick and Haslam (2012; e.g., “Do you get the help you need from other people?”; α = 0.94), to which participants responded using a 7-point scale (1 = *Not at All*, 7 = *Completely*).

##### Well-being

Two measures indexed this construct. A single item of overall well-being, indexing happiness, was used. Previous research has argued that single-item measures of happiness are not only valid but also, produce similar findings to multi-item scales of this construct (see Abdel-Khalek, 2006; Jetten et al., 2012). Participants were asked to respond to the item “Presently would you describe yourself as:” using a 5-point scale from 1 “*Very unhappy*,” to 5, “*Very happy*.”

The Center for Epidemiologic Studies Depression Scale (CES-D; Radloff, 1977) comprising 20 items (e.g., “I was bothered by things that usually don't bother me”) assessed levels of depression (α = 0.91). Each item was rated on a 4-point scale (0 = *Rarely or none of the time*, to 3 = *Most or all of the time*).

### Results

Descriptive statistics for the sample by culture are provided in Table 2. While multiple group membership was not associated with happiness (*r* = −0.058, *p* = 0.657) in Asian participants, it was associated with greater levels of happiness (*r* = 0.343, *p* = 0.002) in Western participants. The difference between these correlations was statistically significant, *Z* = −2.36, *p* = 0.018.

**Table 2:** **Descriptive statistics as a function of culture**.

**Variable**	**Western participants**		**Asian participants**	
	***M***	***SD***	***M***	***SD***
1. Multiple group membership	5.25	1.45	4.84	1.35
2. Well-being: happiness	4.12	0.93	3.50	0.91
3. Well-being: depression	0.76	0.52	0.87	0.45
4. Social support	5.68	1.25	5.28	1.02

*Study 2; N = 137*.

Results also revealed that whereas multiple group membership was not associated with depression (*r* = 0.063, *p* = 0.632) in Asian participants, belonging to multiple groups was associated with lower levels of depression (*r* = −0.317, *p* = 0.005) in Western participants. The difference between these correlations was also statistically significant, *Z* = 2.22, *p* = 0.026.

#### Culture moderates the positive effect of multiple group membership on well-being

**Figure 2:**
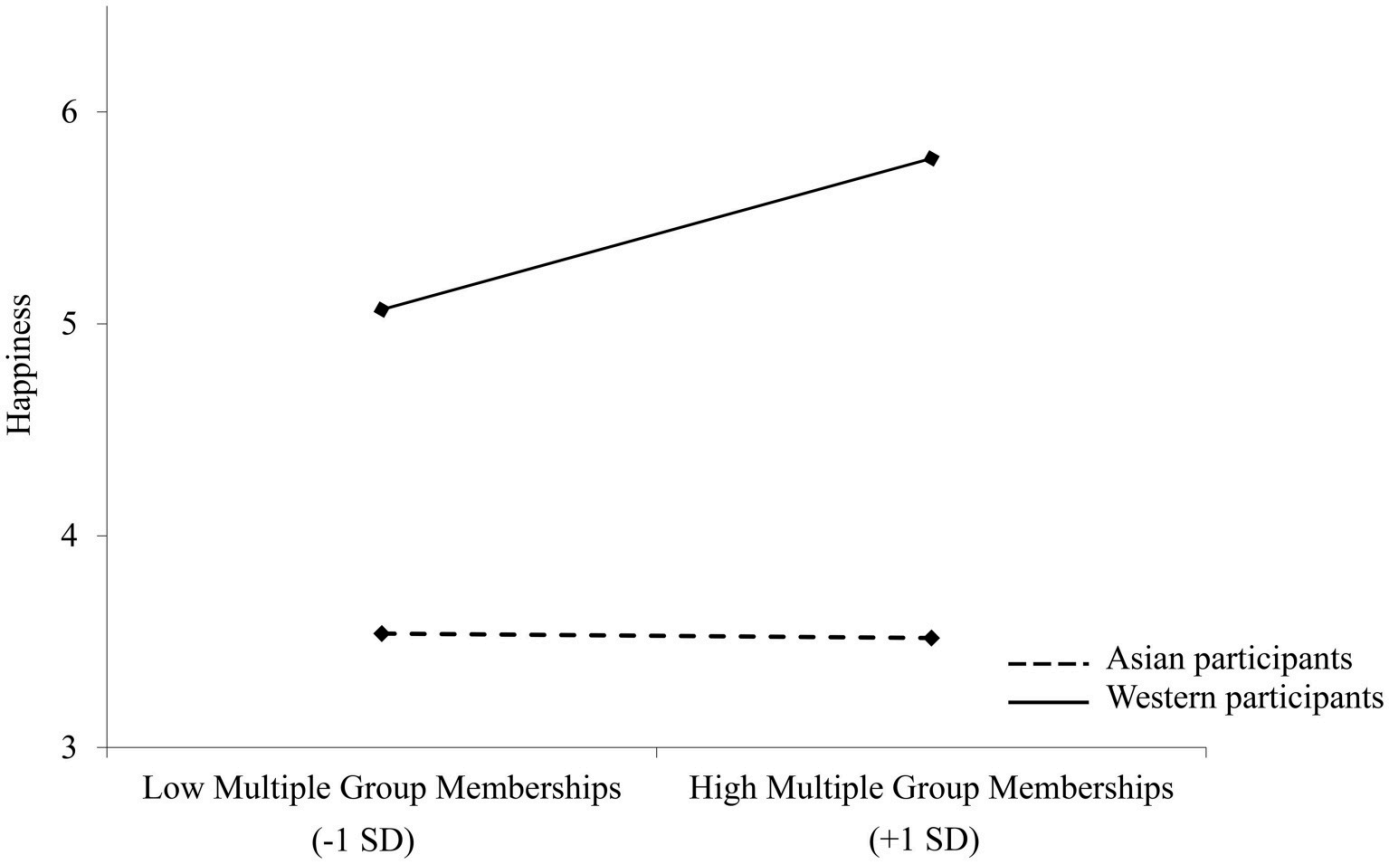
**Happiness ratings as a function of multiple group membership and culture in Study 2; *N* = 137**.

A moderation analysis [Hayes, 2013, model 1; significance levels were calculated using unstandardized values in Hayes PROCESS model 1, as recommended by Hayes and Preacher (2014)] was conducted to examine the influence of culture as a moderator of the relationship between multiple group membership and happiness. Multiple group membership was included as a continuous predictor, with happiness as the outcome variable. Culture (Asian vs. Western) was entered as the moderator. Results showed that culture, *B* = 0.29, 95% CI [0.131, 0.456], *t* = 3.57, *p* < 0.001, but not multiple group membership, *B* = 0.11, 95% CI [−0.019, 0.233], *t* = 1.67, *p* = 0.097, significantly predicted happiness. The interaction between culture and multiple group membership was significant, *B* = 0.13, 95% CI [0.006, 0.254], *t* = 2.07, *p* = 0.041. Simple slopes analysis revealed that multiple group membership was associated with higher levels of happiness for Western participants, *B* = 0.22, 95% CI [0.041, 0.400], *t* = 2.42, *p* = 0.017, but not for Asian participants, *B* = −0.04, 95% CI [−0.211, 0.132], *t* = −0.45, *p* = 0.650 (see Figure 2).

**Figure 3:**
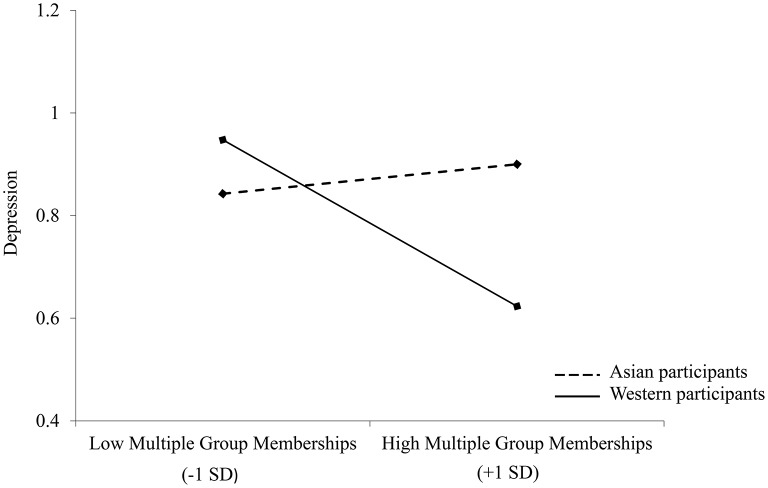
**Depression ratings as a function of multiple group membership and culture in Study 2; *N* = 137**.

A second moderation analysis was conducted to examine the influence of culture as a moderator of the relationship between multiple group membership and depression. Analysis revealed no significant effect of culture, *B* = −0.04, 95% CI [−0.128, 0.042], *t* = −1.01, *p* = 0.314, or multiple group membership, *B* = −0.06, 95% CI [−0.122, 0.012], *t* = −1.62, *p* = 0.107, on depression. However, the interaction between culture and multiple group membership was significant, *B* = −0.07, 95% CI [−0.135, −0.0008], *t* = −2.00, *p* = 0.047. Simple slopes analysis revealed that multiple group membership was associated with lower levels of depression for Western participants, *B* = −0.11, 95% CI [−0.208, −0.021], *t* = −2.42, *p* = 0.017, but not for Asian participants, *B* = 0.02, 95% CI [−0.075, 0.117], *t* = 0.43, *p* = 0.665 (see Figure 3).

#### Social support mediates the positive effect of multiple group membership on well-being for Western, but not Asian, participants

**Figure 4:**
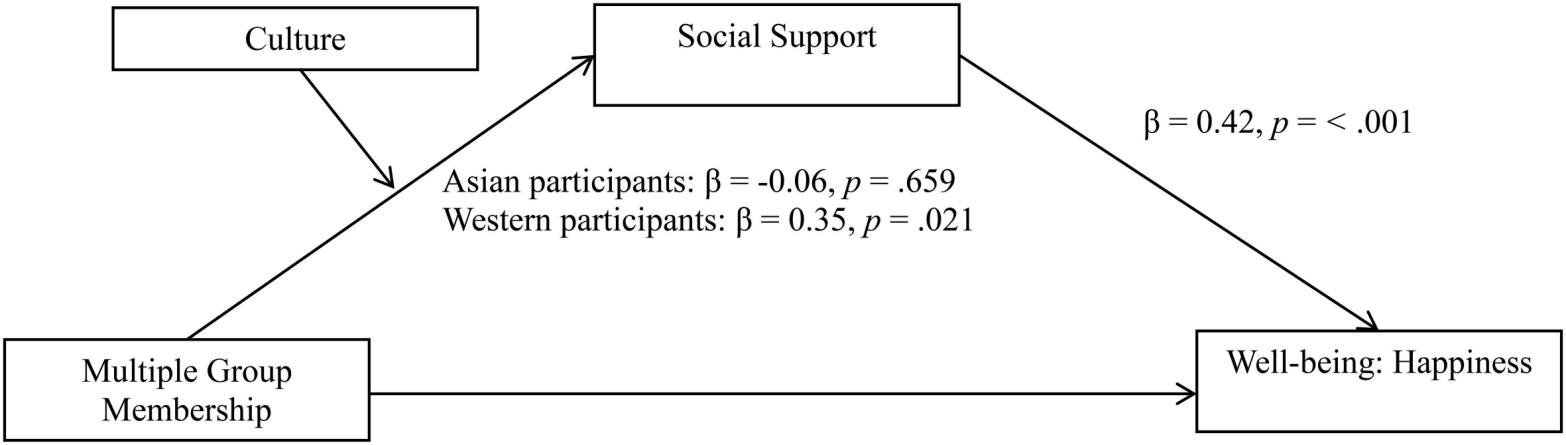
**The relationship between multiple group membership and happiness is mediated through social support, but only among Western participants**. Study 2; *N* = 137. Standardized beta values are reported in the figure to aid interpretability, however, unstandardized coefficients were used to assess significance (as reported in the text), in accordance with recommendation (Hayes and Preacher, 2014).

To test whether the positive effect of multiple group membership on happiness could be explained by a difference between cultural groups in the mediating role of social support, a moderated mediation analysis with 10,000 bootstrap samples was conducted [Hayes, 2013, model 8; significance levels were calculated using unstandardized values in Hayes PROCESS model 8, as recommended by Hayes and Preacher (2014)]. Multiple group membership was included as a continuous predictor, with happiness as the outcome variable. Social support was entered as a continuous mediator, and culture (Asian vs. Western) was entered as the moderator. Analysis revealed no significant effect of multiple group membership, *B* = 0.14, 95% CI [−0.031, 0.307], *t* = 1.61, *p* = 0.109, or culture, *B* = 0.18, 95% CI [−0.013, 0.374], *t* = 1.85, *p* = 0.067, on social support. However, the interaction between multiple group membership and culture on social support was significant, *B* = 0.17, 95% CI [0.002, 0.335], *t* = 2.00, *p* = 0.048. Conditional indirect effects (IE) revealed a significant indirect effect of multiple group membership on happiness via social support for Western participants, IE = 0.10, standard error [SE] = 0.05, 95% CI [0.026, 0.205], but not for Asian participants, IE = −0.02, standard error [SE] = 0.04, 95% CI [−0.107, 0.059]. Results showed that the indirect effect through social support was significantly different between Asian and Western participants, *B* = 0.12, 95% CI [0.017, 0.267]. Culture did not moderate the effect of multiple group membership on happiness when the mediator was in the model, *B* = 0.07, 95% CI [−0.032, 0.170], *t* = 1.35, *p* = 0.181 (See Figure 4). This is consistent with our prediction that it is the capacity of MGM to provide support that is moderated by culture, and suggests that this mechanism fully explains the cultural differences in the relationship between MGM and well-being.

**Figure 5:**
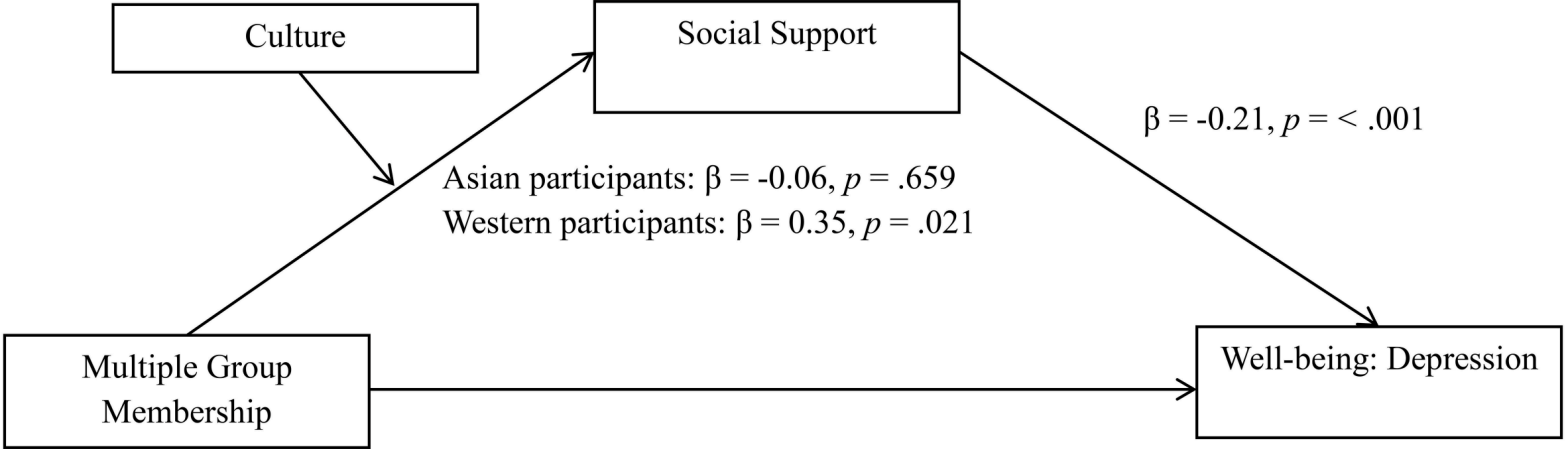
**The relationship between multiple group membership and depression is mediated through social support, but only among Western participants**. Study 2; *N* = 137.

To examine whether social support would mediate the effect of multiple group membership on depression for Western, but not Asian, participants, a second moderated mediation analysis with 10,000 bootstrap samples was conducted (Hayes, 2013, model 8). The interaction between multiple group membership and culture on social support was significant, *B* = 0.17, 95% CI [0.002, 0.335], *t* = 2.00, *p* = 0.048. Conditional indirect effects (IE) revealed a significant indirect effect of multiple group membership on depression via social support for Western participants, IE = −0.05, standard error [SE] = 0.02, 95% CI [−0.104, 0.013], but not for Asian participants, IE = 0.01, standard error [SE] = 0.02, 95% CI [−0.029, 0.055]. Results showed that the indirect effect through social support was significantly different between Asian and Western participants, *B* = −0.06, 95% CI [−0.136, −0.008]. Culture did not moderate the effect of multiple group membership on depression when the mediator was in the model, *B* = −0.04, 95% CI [−0.094, 0.020], *t* = −1.28, *p* = 0.202 (See Figure 5).

We also tested the most plausible alternative mediation model, which was that individuals with better well-being would be more likely to engage in drawing social support from different social groups, and thus, more likely to feel a part of multiple groups (Hayes, 2013, model 4). Happiness was included as a continuous predictor, with multiple group membership as the outcome variable. Social support was entered as a continuous mediator. However, this model was not consistent with the data. In the Western subsample, both the indirect effects (IE) of happiness, IE = 0.19, standard error [SE] = 0.16, 95% CI [−0.070, 0.562], and depression, IE = −0.34, standard error [SE] = 0.22, 95% CI [−0.855, 0.035], on multiple group membership were not significant. Similarly, in the Asian subsample, both the indirect effects of happiness and depression on multiple group membership were not significant.

### Discussion

In Study 2, we found that belonging to multiple groups was associated with greater levels of psychological well-being for Western participants, but not for Asian participants, and this was true across two indexes of well-being: happiness and depression. More specifically, we found that only for Western participants, the impact of belonging to multiple groups on well-being was fully mediated by social support.

It thus appears that there are boundary conditions for the effect of MGM on well-being. Even though MGM serve as a psychological resource from which individuals can receive and benefit from the support provided by fellow group members to enhance well-being (Haslam, 2004; Jetten et al., 2012, 2014), for Asian participants, we observed that they were not deriving as much support from their group memberships, reducing the positive effect of multiple group membership on well-being. We propose that this lack of social support from MGM arises from Asian cultural norms about relationships and support seeking in which there is a reluctance to seek support because one should not burden their social networks (Taylor et al., 2004; Kim et al., 2006, 2008). We test this prediction in Study 3 by including measures indexing concerns about burdening others.

A potential limitation of Studies 1 and 2 is that the studies' samples comprised Asian international students who find themselves in a unique situation. They might belong to many different groups in their home countries (e.g., family, friendship, community, recreational groups), but experience difficulty in utilizing them for support resources and deriving associated well-being benefits because they are not easily accessible (i.e., located overseas in their home countries). Also, the group memberships to which Asian international students belong in Australia may be quite different from those of Australians. That is, the new group memberships may be weaker, less stable or less meaningful given they have only been part of them for a shorter duration; possibly limiting the extent to which support resources can be derived from these memberships. This may explain why Asians may derive fewer well-being benefits from multiple groups relative to Westerners in our studies. We address this limitation in Study 3.

## Study 3

In Study 3, we measured perceived burden as a proxy to assess one's reluctance to draw upon relationships for support resources. In line with the above reasoning, we tested whether the effect of MGM on well-being was dependent on the extent to which one is reluctant to seek social support from their relationships. Specifically, we expected that MGM would only be associated with enhanced psychological well-being for those Asian participants who were least reluctant to draw on support from others. Importantly, Study 3 also addressed a limitation of Studies 1 and 2 by investigating this prediction in a sample of Asian students at a university in Singapore residing within their cultural context.

### Method

#### Participants

Participants were 105 students at a Singapore university (43 males and 62 females). Participants received 6 Singapore dollars for their participation. On average, participants were 21.4 years old (*SD* = 1.54) and all described their ethnicity as Chinese.

#### Measures

Participants completed a survey that included demographic questions, the multiple group membership scale (see Study 2; Jetten et al., 2010; *r* = 0.59.), and the Satisfaction with Life Scale (see Study 1; Diener et al., 1985; α = 0.85).

##### Reluctance to enlist social support

Three items were developed to measure the extent to which one is reluctant to enlist social support from relationships due to concerns about burdening others (i.e., “It is important for me not to burden others with my problems”; “One should avoid troubling others,” and “I make an effort not to impose on others”; α = 0.80). Each item was rated on a 7-point scale (1 = *Strongly disagree*, 7 = *Strongly agree*).

### Results and discussion

Table 3 provides descriptive statistics and results of bivariate correlations between key variables. Multiple group membership (*M* = 5.14, *SD* = 1.07) was not significantly related to life satisfaction (*M* = 4.57, *SD* = 1.12), *r* = 0.130, *p* = 0.186.

**Table 3:** **Descriptive statistics and correlations**.

**Variable**	***M***	***SD***	**1**	**2**	**3**
1. Multiple group membership	5.14	1.07	–	0.17	0.13
2. Reluctance to enlist support	5.23	1.03		–	0.04
3. Well-being: Life satisfaction	4.56	1.12			–

*Study 3; N = 105*.

^*^p < 0.05, ^**^p < 0.01.

**Figure 6:**
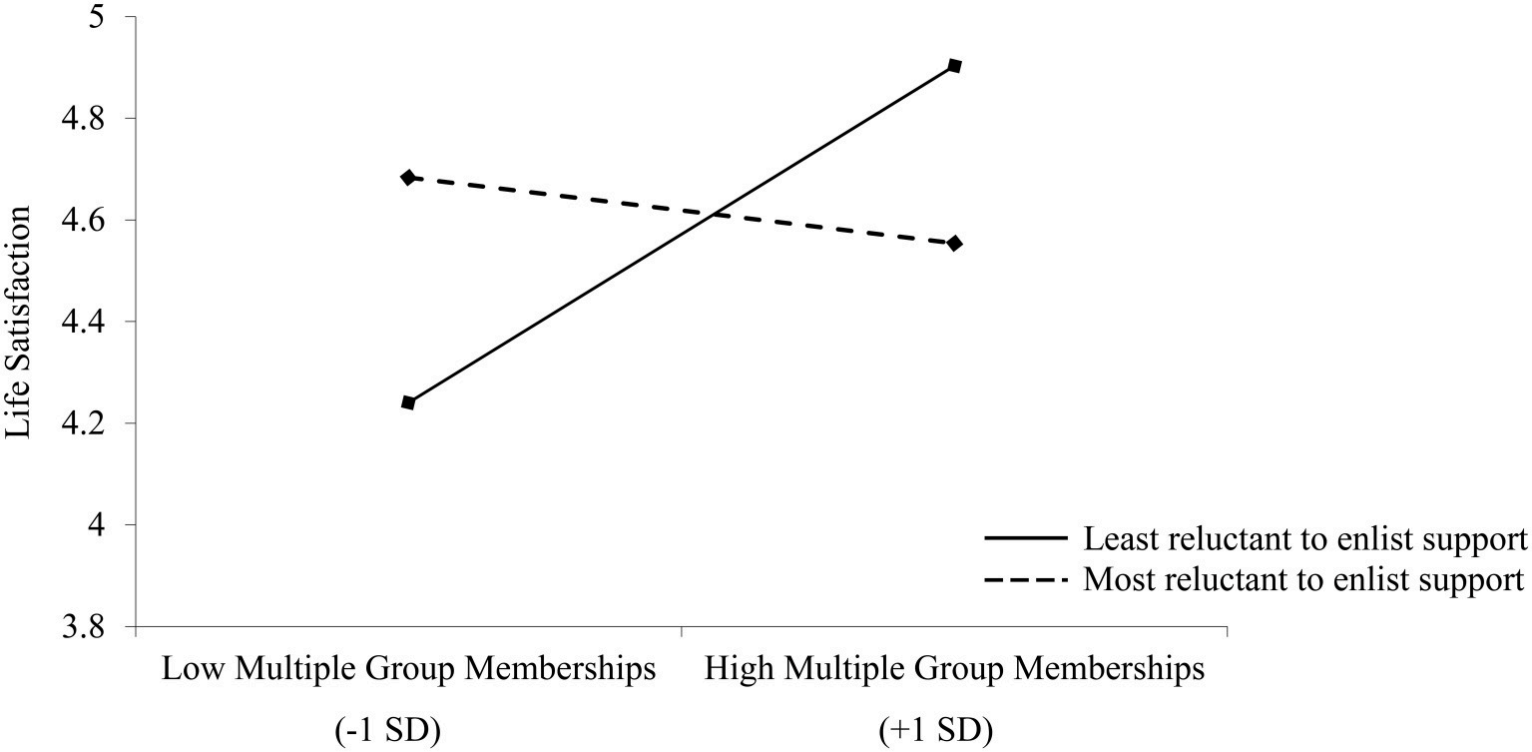
**Life satisfaction ratings as a function of multiple group membership and one's reluctance to enlist social support in Study 3; *N* = 105**.

A moderation analysis (Hayes, 2013, model 1; significance levels were calculated using unstandardized values) was used to examine whether the degree of reluctance to enlist social support would moderate the relationship between multiple group membership and life satisfaction. Multiple group membership was included as a continuous predictor, and life satisfaction as the outcome variable. Reluctance to enlist social support was entered as a continuous moderator. There was no significant effect of multiple group membership, *B* = 0.13, 95% CI [−0.072, 0.332], *t* = 1.28, *p* = 0.205, or reluctance to enlist support, *B* = 0.02, 95% CI [−0.208, 0.252], *t* = 0.19, *p* = 0.850, on life satisfaction. Consistent with our prediction, the interaction term between belonging to multiple groups and reluctance to enlist support was significant, *B* = −0.18, 95% CI [−0.352, −0.010], *t* = −2.10, *p* = 0.038. Simple slopes analysis revealed that belonging to multiple groups was associated with greater levels of life satisfaction for those who were least reluctant to enlist social support, *B* = 0.32, 95% CI [0.034, 0.597], *t* = 2.22, *p* = 0.029, but was unrelated to life satisfaction for participants who were more reluctant to enlist social support, *B* = −0.06, 95% CI [−0.308, 0.197], *t* = −0.44, *p* = 0.663 (see Figure 6).

Here, we replicate the findings from previous studies that MGM are not associated with psychological well-being for Asian participants. However, additionally, we found that one's reluctance to enlist social support moderated the relationship between multiple group membership and psychological well-being, highlighting that belonging to multiple groups was in fact, associated with greater well-being among those Asian participants who were less reluctant to enlist social support. As the findings from our moderation analysis suggest, belonging to multiple groups may benefit the well-being of Asians to a lesser extent, especially when they are more sensitive to concerns about burdening others. This potentially increases their reluctance to draw on support resources from their relationships, reducing the positive effect of MGM on well-being.

Taken together, the findings across the three studies show that for Asians, multiple group membership is not strongly associated with psychological well-being, with correlations ranging between 0.058 and 0.130 across three studies and five indicators of well-being. This suggests that for Asians, the relationship between multiple group membership and well-being is weaker than it is for Westerners. To further interrogate this point, we conducted an empirical review of the work assessing the relationship between MGM and well-being.

## Study 4

Study 4 aimed to (1) investigate the robustness of the effect of MGM on well-being in the literature (both the Asian and Western cultural contexts), and (2) determine whether this effect is smaller in Asian, compared to Western, cultural contexts. Addressing these aims, we obtained effect sizes by identifying and integrating relevant studies in the literature examining the relationship between MGM and psychological well-being. PsycINFO and PubMed were searched for relevant studies using the following keywords: *multiple group membership, social group memberships*, and *greater number of group identification*. Eligible studies were published between 1990 and August 2015, in English and in a peer-reviewed journal, and included quantitative measures or manipulations of number of group memberships across different life domains (e.g., leisure or social group, community group, family) along with a dependent quantitative measure of psychological well-being (i.e., life satisfaction, happiness, self-esteem, psychological distress, mood, depression, anxiety, and stress).

The search produced a total of 735 references in PsycINFO and 1030 references in PubMed, of which 1564 were unique references. Of these, we identified 15 relevant articles reporting 23 different studies, with a total of 14,063 participants. None of these studies compared Westerners with Asians, and only four were conducted with Asian participants. More detail on each of the studies is provided in Table 4. In addition to these studies, we included our data sets from Study 1 to Study 3 in the integrative analysis. This resulted in 27 independent studies for the analysis.

**Table 4:** **The relationship between multiple group memberships and psychological well-being, as reported in 27 studies**.

**No**.	**References**	**Journal**	**Study**	**N**	**Population**	**Multiple group membership measure**	**Psychological well-being measure**	**Overall well-being effect size (*r*)**	**Country**
**ASIANS**									
1			1	180	Students	4-item exeter identity transition scales Haslam et al., 2008	Satisfaction with life scale (Diener et al., 1985) and DASS-21 Lovibond and Lovibond, 1995	+0.10	International students born in Asia
2			2	60	Students	2-item scale (Jetten et al., 2010)	1-item happiness scale and CES-D (Radloff, 1977)	−0.06	International students born in Asia
3			3	105	Students	2-item scale (Jetten et al., 2010)	Satisfaction with Life Scale (Diener et al., 1985)	+0.13	Singapore
4	Ganga et al., 2014	Mental Health Review Journal		453	Young adults	Membership in clubs and organizations	The Achutha Menon Centre Positive Mental Health Scale (Ganga and Kutty, 2015)	+0.10^a^	India
5	Jetten et al., 2015	PLoS One	1b	109	Retired older adults	3-item exeter identity transition scales (Haslam et al., 2008)	1-item personal self-esteem adapted from the quality of life questionnaire (Logsdon et al., 2002)	+0.23	China
6			3a	154	Students	4-item scale	3-item personal identity strength adapted from Baray et al. (2009)	+0.19	China
7	Takahashi et al., 2011	BMC Public Health		92	Individuals with musculoskeletal impairments	Adapted social capital assessment tool (Harpham et al., 2006)	Satisfaction with Life Scale (Diener et al., 1985)	+0.26^a^	Vietnam
**WESTERNERS**									
8	Brook et al., 2008	Personality and Social Psychology Bulletin		372	Students	Getting participants to list all of their important identities (i.e., group memberships and social roles)	CES-D (Radloff, 1977), anxiety subscale (Bradley and Lewis, 1990), well-being subscale (Bradley and Lewis, 1990), and Perceived Stress Scale (Cohen et al., 1983)	+0.08	United States—66.7% White, 12.9% Asian American, and 8.1% African American
9			2	77	Students	2-item scale (Jetten et al., 2010)	1-item happiness scale and CES-D (Radloff, 1977)	+0.33	Australia
10	Cruwys et al., 2015	Social Psychological and Personality Science	1	139	Students	7-item exeter identity transition scales (Haslam et al., 2008)	CES-D (Radloff, 1977)	+0.28	Australia
11			2	88	Students	Experimental manipulation of social identity salience by getting individuals to reflect on no groups, one group, or three groups	The positive and negative affect scale (Watson et al., 1988)	+0.24	Australia
12	Haslam et al., 2008	Neuropsychological Rehabilitation		53	Recovering stroke patients	12-item exeter identity transition scales (Haslam et al., 2008)	Life satisfaction scale (Haslam et al., 2005) and Chronic stress scale (Haslam and Reicher, 2006)	+0.21	United Kingdom
13	Haslam et al., 2011	British Journal of Psychology		32	Older adults	3-item exeter identity transition scales (Haslam et al., 2008)	5-item personal identity strength scale (Jetten et al., 2010)	+0.44^a^	United Kingdom
14	Iyer et al., 2009	British Journal of Social Psychology		100	Students	6-item exeter identity transition scales (Haslam et al., 2008)	8-item well-being scale (Branscombe et al., 1999)	+0.08	United Kingdom
15	Jetten et al., 2015	PLoS One	1a	29	Children	3-item exeter identity transition scales (Haslam et al., 2008)	7-item self-esteem scale adapted from Rosenberg (1965)	+0.48	United Kingdom
16			2	813	Adolescents	3-item exeter identity transition scales (Haslam et al., 2008)	1-item self-esteem scale (Robins et al., 2001)	+0.29	Australia
17			3b	78	Students	3-item exeter identity transition scales (Haslam et al., 2008)	1-item self-esteem scale (Robins et al., 2001)	+0.42	Australia
18			4	302	Students	By determining how many of the three identities (i.e., gender, university sports team fan, nationality) participants rate as being of more than median-level importance	Rosenberg Personal Self-esteem scale (Rosenberg, 1965)	+0.25	United States
19			5	148	Students	By assessing the extent to which participants rate the importance of seven identities as higher than the median	Rosenberg personal self-esteem scale (Rosenberg, 1965)	+0.20	United States
20	Jetten et al., 2013	Social Psychological and Personality Science		816	Students	2-item exeter identity transition scales (Haslam et al., 2008)	Satisfaction with life scale (Diener et al., 1985)	+0.23	United Kingdom
21	Johnstone et al., 2015	Frontier of Psychology		76	Individuals residing in homelessness accommodation services	2-item scale (Jetten et al., 2010) and 4-item exeter identity transition scales (Haslam et al., 2008)	Personal well-being index by the international well-being group (2006)	+0.39	Australia
22	Jones et al., 2012	British Journal of Health Psychology		93	Patients with orthopadic injuries or acquired brain injuries	12-item Exeter Identity Transition Scales (Haslam et al., 2008)	General health questionnaire (Goldberg, 1992)	+0.14	United Kingdom
23	Murray et al., 2007	Social Psychiatry and Psychiatric Epidemiology		394	Adults	Total number of groups to which participants belong to	The positive and negative affect scale (Watson et al., 1988) and satisfaction with life scale (Diener et al., 1985)	+0.13	Australia
24	Sani et al., 2015	Social Psychiatry and Psychiatric Epidemiology		1800	Adult general practitioner attendees	Group identification scale (Sani et al., 2014)	Major depression inventory (Bech et al., 2001)	+0.36	Scotland
25	Witherspoon et al., 2009	Applied Developmental Science		437	Adolescents	Network of relationships inventory (Furman and Buhrmester, 1985), psychological sense of school membership scale (Goodenow, 1993) and 10-item neighborhood connectedness scale	Rosenberg Personal Self-esteem scale (Rosenberg, 1965)	+0.28^a^	United States—29% Chinese American, 26% White, 23% African American, and 11% Puerto Rican
26	Ysseldyk et al., 2013	Aging and Mental Health	1	42	Older adults from structured care homes	8-item exeter identity transition scales (Haslam et al., 2008)	Geriatric depression scale (Sheikh and Yesavage, 1986)	+0.39	Canada
27			2	7021	Older adults—English Longitudinal Study of Aging at Wave 1	Total number of groups to which participants belong to	4-item depression scale	+0.16^a^	United Kingdom

a *Effect sizes obtained were controlled for other variables (e.g., demographics*).

### Results and discussion

The meta-analytical procedure reported in Borenstein et al. (2009) was used to determine the overall relationship between MGM and well-being. From the studies identified, 27 effect sizes were calculated with a total sample size of 14,063 participants and correlations ranging from −0.06 to 0.48. A random effects model (which assumes that the true effect size varies across studies and follows a normal distribution around the mean) revealed an overall mean effect size of *r* = 0.22, 95% CI [0.175, 0.267], *Z* = 9.13, *p* < 0.001.

To compare the effect size of studies consisting primarily of Westerners to studies involving Asians, subgroup analyses were conducted. For the studies with Asian participants (*n* = 7), 7 effect sizes were calculated with a total of 1153 participants and correlations varying between −0.06 and 0.26. The random effects model revealed a mean effect size of *r* = 0.13, 95% CI [0.072, 0.187], *Z* = 4.38, *p* < 0.001, indicating that the effect size was small (Cohen, 1988). For the studies with Western participants (*n* = 20), 20 effect sizes were calculated with a total sample size of 12,910 participants and correlations ranging between 0.08 and 0.48. A random effects model showed a mean effect size of *r* = 0.25, 95% CI [0.194, 0.302], *Z* = 8.67, *p* < 0.001, for these studies. More specifically, in 95% of all possible meta-analyses, the true mean effect would probably fall in the range of 0.194–0.302 for Westerners, indicating a robust effect. In contrast, for Asians, the true mean effect would usually fall in the range of 0.072–0.187, suggesting there is a higher probability that the true effect is weaker for Asians, and likely to be null in some analyses (Cohen, 1988). As it is predicted that multiple group membership processes would be different across cultures, in calculating the *Q*-value, the between-studies variance, *T*_2_, was computed within subgroups and used as a separate estimate for each subgroup. The *Q*-value for the difference was 8.75 with 1 *df* and *p* = 0.003.

The present analysis is the first review applying meta-analytic techniques to interrogate the relationship between MGM and indices of well-being. We found some evidence that multiple group membership was more strongly related to well-being in the studies involving Westerners than in studies involving Asians. This strengthens our argument that individuals in cultural contexts that privilege drawing support from their relationships may reap greater well-being benefits from their MGM.

Nevertheless, some caution is warranted in interpreting the results of this review. The purpose of this review was not to provide an all-encompassing meta-analysis, but rather an initial interrogation of the existing literature applying meta-analytic principles. A potential limitation is the small number of studies included in the Asian subgroup analysis. Of note, Studies 1–3 contributed 3 out of the 7 studies in this analysis, and whilst these all had confidence intervals including 0, the other 4 studies did not. The reason for this is not clear, but publication bias against null effects may play a role (Franco et al., 2014). If so, the mean effect size for Asians in reality may be even weaker, relative to Westerners. Despite this, our findings suggest that compared to Westerners, Asians may generally derive fewer well-being benefits from belonging to multiple groups.

## General discussion

In this current research, we apply a novel theoretical framework to understand some important cross-cultural differences in the relationship between MGM and psychological well-being. Overall, we found support for our argument that, belonging to multiple groups may confer little, or fewer, well-being benefits for Asians relative to Westerners. Importantly, our findings provide further evidence for a growing body of research claiming that MGM can form the basis of a “social cure”; the sense of shared social identities derived from belonging to multiple groups are an important basis for social support, which is a critical social factor in protecting well-being (Haslam et al., 2009; Jetten et al., 2012). Of note though, we provide some of the first evidence for the boundary conditions of this effect, showing that this relationship is more likely to be observed for individuals from Western than Asian cultural contexts.

For Asians, we found that multiple group membership was not significantly, or only weakly, associated with well-being. This was replicated across 3 survey studies in samples comprising Asian students from Australia and Singapore using multiple indices of well-being (Studies 1–3), and was also reflected in the findings of our review applying meta-analytic techniques (Study 4). In particular, in our cross-cultural study (Study 2) and review (Study 4), we showed that belonging to multiple groups was comparatively less beneficial to the psychological well-being of Asians, relative to Westerners. This leads us to conclude that cultural differences in the extent to which individuals derive well-being benefits from belonging to multiple groups can exist. Specifically, whilst belonging to multiple groups is likely to confer well-being benefits for individuals from Western cultures, the “more the merrier” effect may hold less truth for individuals from Asian cultures. As these findings suggest, it is important to consider an individual's cultural context to obtain a full understanding of their psychology (Kim and Markus, 1999).

Critically though, in examining the cultural underpinnings of multiple group membership processes, the present analysis sheds new light on the contribution that cultural background makes to understanding how individuals derive psychological resources from their group memberships in different ways. Utilization of *social group resources* (e.g., support) is in fact interconnected with cultural norms about relationships. Specifically, it was found in Study 2, that social support mediated the effect of multiple group membership on well-being for Westerners but not Asians. Also, Study 3 showed that among Asians, multiple group membership was only associated with well-being for those who were least reluctant to enlist social support due to concerns about burdening others. Our current work therefore provides the first empirical demonstration that, compared to Westerners, Asians may reap fewer well-being benefits from belonging to multiple groups. This is possibly because of an emphasis on different cultural norms on relationships and support seeking across cultures (i.e., the extent to which it is perceived as appropriate to draw support resources from others), thereby leading support seeking to be perceived differently in different cultures (Kim et al., 2008). Among Asians, the cultural notion that it is important not to be a source of burden on others may cause them to worry about imposing on their relationships by requesting support (Kim et al., 2008) which, in turn, strengthens their reluctance to draw on such resources from their MGM resulting in reduced well-being benefits being derived from these memberships. Conversely, the present analysis suggests that individuals from Western cultures appear to have fewer concerns about deriving support from their social group memberships, which in turn is associated with enhanced well-being. In fact, these findings are in line with the literature showing cross-cultural differences in group processes, where it is argued that self-concepts and group behaviors are construed in more relational ways in Asian cultural contexts than in Western cultural contexts (Yuki and Takemura, 2013). In groups, Asians may be more focused on maintaining harmonious and reciprocal relationships, and therefore, they may be more reluctant to seek support from their group members in order to maintain these delicate interpersonal ties. By contrast, maintaining interpersonal ties and social harmony in groups may be less of a concern in Western cultural contexts. In this way, Westerners may feel more able to obtain support from their MGM.

Despite these cultural variations in notions about support seeking, it is noteworthy that Study 2 found social support to be associated with greater well-being for both Asians and Westerners. This finding is in line with an extensive body of research documenting the mental health benefits of social support (House et al., 1988; Thoits, 1995). What is interesting from the findings though, is that while Asian cultural norms about relationships and support seeking can make Asians more reluctant to enlist social support (Taylor et al., 2004; Kim et al., 2006, 2008), this does not appear to prevent them from perceiving such support as beneficial. Specifically, for Asians, the findings suggest that the costs involved in utilizing social support (e.g., highlighting one's incompetence; over-taxing the resources of others; Bolger et al., 2000; Seidman et al., 2006) were more likely to occur *in the process* of deriving support from others, rather than *after* such support was provided. This is reflected in the non-significant relationship between multiple group membership and social support, but significant positive relationship between social support and well-being. In contrast, we did not find evidence of this “cost” in our sample of Westerners. Given that previous research has highlighted that Asians can benefit from seeking and receiving support from relationships which are perceived as mutual and interdependent (i.e., the individual had previously provided support to the relationship partner; Wang and Lau, 2015), future research could examine these processes in greater detail. For example, Asians may possibly derive support resources from their mutual relationships when belonging to multiple groups, thereby reaping well-being benefits from MGM.

We note that the current studies were not without limitations. First, the cross-sectional nature of the data does not permit us to make causal inferences, and thus, directionality of effects cannot be inferred. However, it is worth noting that the alternative model that we tested in Study 2 did not provide a superior fit for the data. Also, because our study samples comprised only university students (Studies 1–3), the findings may not generalize to a non-university population or to clinical populations who may be more socially isolated. All these limitations should be addressed in future work by examining the generalizability of findings to other populations and by examining evidence for the predicted relationship longitudinally.

Another limitation is that our hypotheses in Studies 1 and 2 (i.e., weaker relationship between MGM and well-being for Asians, relative to Westerners) were examined using samples of Asian international students. Directly comparing Asian international students to Australian Caucasian students may have created a potential confound in Study 2—that these Asian participants' unique situation may have influenced the ease and extent to which they derived support and gained well-being benefits from their group memberships. The present data do not allow us to rule out this alternative explanation. However, our findings from Study 3 speak to this potential confound. In this study, we replicated the findings with a sample of Asian students in Singapore who were within the context of their home culture, and showed that multiple group membership was not associated with well-being. This finding, obtained in an un-confounded study context, substantiates our argument that Asians may generally derive fewer well-being benefits from belonging to multiple groups than Westerners. Nevertheless, it is recommended that future research explore and replicate these effects in other samples, for instance, (a) Asians who are within their cultural context or (b) Asian international students could be compared to Western international students (e.g., Europeans) in the Australian context.

Lastly, for practical reasons, social support was measured using four items adapted from Van Dick and Haslam (2012) instead of the much longer COPE Inventory (Carver et al., 1989), a commonly used measure to assess cultural differences in social support seeking (Kim et al., 2006). This social support measure has in fact been shown to demonstrate high reliability in Western samples (Van Dick and Haslam, 2012), though it has not previously been used in Asian samples. Apart from this, the other measures used in our studies have previously been used in both Asian and Western samples with adequate psychometric properties. In general, nonetheless, it is recommended that future work should examine the hypotheses using other scales to extend as well as replicate our findings.

Overall, our findings are consistent with the literature demonstrating cross-cultural differences in group processes (e.g., variations across cultures in how individuals relate to groups; Yuki and Takemura, 2013). However, there are several possible interpretations of our findings. For instance, research has suggested that MGM lower one's well-being because groups can demand too much effort and deplete one's limited time and energy (Bolger et al., 1989). In line with the strong motivation to maintain harmonious and reciprocal relationships, and relatedly, the strong emphasis on the fulfillment of role-based obligations in relationships in Asian cultural contexts (Rothbaum et al., 2000), it is therefore possible that Asians derive fewer well-being benefits from belonging to multiple groups because of their need to fulfill obligations (e.g., to reciprocate support). This explanation should be examined in future work, ideally with populations other than undergraduate students. In addition, future research could examine these cross-cultural differences in group processes in greater detail to understand how people conceptualize their MGM. This may help to shed light on cultural differences in what constitutes MGM and what it means to belong to multiple groups.

Nevertheless, the current work has generated important insights about the function of multiple group membership processes in different cultural contexts. More specifically, it has provided the first empirical evidence on the circumstances in which these group memberships can sometimes fail to have any positive effects on well-being. This has important practical implications for psychosocial interventions involving attempts to enhance well-being by (1) advocating the building and development of social group memberships or (2) facilitating individuals to draw support resources from their group memberships (Jetten et al., 2014; Newlin et al., 2015). Our findings suggest that it may be important to tailor these interventions to the needs of Asians. For instance, when working with Asian clients, therapists may need to work with clients' reluctance to draw upon support resources from their relationships and help them tap these resources in a way that is consistent with Asian culture normative standards.

### Conclusion

Although the relationship between MGM and psychological well-being appears to be relatively well-established, our findings indicate that there is cross-cultural variation in this association. The well-being benefits from MGM are not only determined by how much support can be derived from belonging to multiple groups, but also how easy it is for an individual to draw upon these resources. Given that Asian cultural norms prescribe individuals not to draw on such support lightly, unfortunately, Asians may not fully benefit from the psychological resources their group memberships encompass.

## Author contributions

MC, JJ, TC, and CH contributed to the study design. MC and NP collected the data. MC performed the data analysis and interpretation under the supervision of JJ, TC, and CH. MC drafted the manuscript, and JJ, TC, and CH provided critical revisions.

### Conflict of interest statement

The authors declare that the research was conducted in the absence of any commercial or financial relationships that could be construed as a potential conflict of interest.
